# Branched Polyethylenimine-Superparamagnetic Iron Oxide Nanoparticles (bPEI-SPIONs) Improve the Immunogenicity of Tumor Antigens and Enhance Th1 Polarization of Dendritic Cells

**DOI:** 10.1155/2015/706379

**Published:** 2015-06-28

**Authors:** My-Dung Hoang, Hwa-Jeong Lee, Hyun-Ju Lee, Sung-Hoon Jung, Nu-Ri Choi, Manh-Cuong Vo, Thanh-Nhan Nguyen-Pham, Hyeoung-Joon Kim, In-Kyu Park, Je-Jung Lee

**Affiliations:** ^1^Research Center for Cancer Immunotherapy, Chonnam National University Hwasun Hospital, Hwasun, Jeollanamdo 519-763, Republic of Korea; ^2^Department of Hematology-Oncology, Chonnam National University Hwasun Hospital, Hwasun, Jeollanamdo 519-763, Republic of Korea; ^3^Department of Biomedical Science, Chonnam National University Medical School, Gwangju 500-872, Republic of Korea

## Abstract

Nanoparticles in the field of dendritic cell (DC) research are emerging as a promising method of enhancing the efficacy of cancer immunotherapy. We investigated the effect of branched polyethylenimine-superparamagnetic iron oxide nanoparticles (bPEI-SPIONs) on tumor cells loaded onto DCs. The tumor antigens were prepared as follows: (1) apoptotic U266 cells with ultraviolet B (UVB) irradiation followed by a 2 h incubation in the absence (2 h postirradiated cells) or (2) presence of bPEI-SPIONs (bPEI-SPION 2 h postirradiated cells) and (3) apoptotic U266 cells with UVB irradiation followed by an overnight 16 h incubation (16 h postirradiated cells). bPEI-SPIONs render U266 cells sensitive to UVB irradiation through reactive oxygen species production to accelerate apoptotic death. The 2 h postirradiated cells and bPEI-SPION 2 h postirradiated cells released immunogenic proteins, including Hsp70, Hsp90, and HMGB1. The DCs loaded with bPEI-SPION 2 h postirradiated cells showed the highest IL-12p70 production and Th1 polarization compared with other DCs. These results suggest that bPEI-SPIONs are a promising method of enhancing the immunogenicity of tumor cells and promoting Th1 polarization of DCs loaded with these tumor cells.

## 1. Introduction

Lack of specific hallmark of cancer is reason for using of whole tumor cells (tumor apoptotic bodies, tumor cell lysates, or tumor cell-derived RNA), which represent full characteristics of tumor identity, as common source of tumor antigens in clinical trials of dendritic cell (DC) based cancer vaccines [1, 2]. Among these antigen preparation procedures, ultraviolet B (UVB) irradiation is a safe, inexpensive, and easy method of inducing a mixed population of viable, early apoptotic, and late apoptotic/necrotic cells with various proportions during tumor antigen preparation [3, 4]. However, the immunogenic properties of prepared tumor antigens depend on the cell death stage. Engulfment of the early apoptotic body leads to silent phagocytosis with anti-inflammatory activity, whereas phagocytes are activated when encountering late apoptotic/necrotic cells; as a result, the latter gives rise to an inflammatory response [5, 6]. In our previous studies, apoptotic cells or dying tumor cells, used as a tumor antigen source, showed high antitumor induction efficacy of DCs to T cells [7, 8]. To develop novel techniques for tumor antigen preparation, we induced immunogenic cell death using JSI124 combined with bortezomib in multiple myeloma (MM) [4].

Recently, superparamagnetic iron oxide nanoparticles (SPIONs) have been reported to enhance reactive oxygen species (ROS) production [9]. Based on our previous studies on DCs, we suppose that SPIONs accelerate tumor cell death to an immunogenic induction stage; hence, the antigen can be more highly immunogenic than UVB irradiated tumor antigens. SPIONs are an interesting tool for cell labeling, cell therapy, and diagnostic imaging. However, uncoated SPIONs can cause toxicity to living cells, and coating materials have been developed to stabilize aqueous SPION suspensions and reduce toxicity [10]. Branched polyethylenimine- (bPEI-) SPIONs, iron oxide nanoparticles coated with bPEI, are less toxic than SPIONs and readily bind to the cell membrane to enhance their uptake [11].

Here, we investigated the immunogenicity of tumor antigen sources prepared from UVB irradiated tumor cells in the presence of bPEI-SPIONs during T cell responses elicited by DCs loaded with these tumor antigens. We showed that bPEI-SPIONs accelerated UVB irradiated cell death to the late apoptotic/necrotic stage after 2 h incubation. Furthermore, prepared antigen with bPEI-SPIONs induced the highest production of IL-12p70 of DCs, and these DCs favored Th1 polarization during the T cell response.

## 2. Materials and Methods

### 2.1. Synthesis and Characterization of bPEI-SPION

bPEI-SPION was synthesized by conjugation of low molecular weight bPEI (Mw 1,800 Da, Aldrich) onto thermally cross-linked SPION (TCL-SPION) via amide linkage [12]. The physical-chemical properties of bPEI-SPION were further characterized by using Zetasizer Nano Z (Malven Instruments, Malvern, UK), the transmission electron microscopy (TEM) (JEOL JEM-2000 FXII, Japan), and TGA analysis (Mettler-Toledo, SDT851, Columbus, USA) in order to confirm its successful synthesis.

### 2.2. Intracellular Ferric Iron Measurement

bPEI-SPION uptake by the U266 MM cell line was evaluated using a quantitative spectrophotometric method [13]. Briefly, 5 × 10^5^ U266 cells were put in contact with different amount of bPEI-SPIONs with shaking for 1 h at room temperature. Cells were collected and washed three times in 1x phosphate-buffered saline (PBS) (Sigma Aldrich, St. Louis, MO, USA). The pellet was resuspended in 30% HCl (Sigma Aldrich) for 2 h at 60°C. Next, 0.08% potassium persulfate, 8% potassium thiocyanate, and 3.6% HCl (Sigma Aldrich) were added to form the iron-thiocyanate complex. The absorbance at 490 nm was measured using a microplate reader (TECAN Infinite M200 PRO, Tecan, Männedorf, Switzerland) after 10-min incubation. Aqueous FeCl_3_·6H_2_O (Sigma Aldrich) solution was treated in the same manner to create the standard curve.

### 2.3. Confocal Microscopy

U266 cells were put in contact with bPEI-SPION conjugated with FNR-675 dye (BioActs, Namdong-gu, Incheon, Korea), which appears as a red color under confocal microscopy (Carl Zeiss, Jena, Germany). Cells were fixed on a glass slide and the nuclei were stained with DAPI (Thermo Scientific Pierce, Rockford, USA), which appears as a blue color.

### 2.4. Assays of ROS Generation

2′,7′-Dichlorofluorescein-diacetate (DCFH-DA) (Sigma Aldrich) and N-acetylcysteine (NAC) (Sigma Aldrich), which blocks ROS production, were used to determine intracellular ROS levels based on fluorescence measurements. Briefly, cells were incubated in warm RPMI-1640 medium (Invitrogen Life Technologies, Carlsbad, CA, USA) containing 10% fetal bovine serum (FBS) (PAA, Murarrie, Australia) and 1% penicillin/streptomycin (P/S) (Lonza, Walkersville, MD, USA) with 6 *μ*M DCFH-DA at 37°C for 1 h [14]. The probe was then removed and cells were used for preparation of several types of antigen. ROS levels for each type of antigen were then measured using flow cytometry with a FACS Calibur instrument (Becton Dickinson, Mountain View, CA, USA) at 488 nm. The data were analyzed using WinMDI, version 2.9 (Bio-Soft Net).

### 2.5. Generation of Monocyte-Derived DCs

Monocytes were isolated from peripheral blood mononuclear cells (PBMCs) obtained from healthy donors using a CD14^+^ magnetic activating cell sorting (MACS) system (Miltenyi Biotec Inc., Auburn, CA, USA) and were cultured at 2 × 10^6^ cells/well in six-well plates (BD Falcon, San Jose, CA, USA) in IMDM with L-glutamine and 25 mM HEPES buffer (Gibco-BRL, Grand Island, NY, USA) supplemented with 10% FBS and 1% P/S in the presence of recombinant human IL-4 (50 ng/mL, Peprotech, Rocky Hill, NJ, USA) and GM-CSF (20 ng/mL, Peprotech). Cell culture medium was replenished every 2 days. At day 6, 2 × 10^5^ immature DCs/well were matured with 50 ng/mL TNF-*α* (Peprotech) in 24-well plates (BD Falcon). Two hours after TNF-*α* stimulation, DCs were loaded with several types of tumor antigen.

### 2.6. Tumor Antigen Preparation

Three types of tumor antigen were prepared, as follows: (1) 2 h* postirradiated* antigen-containing U266 cells, which were induced by high-dose UVB irradiation (120 mJ/cm^2^) (International Light, Newburyport, MA, USA) followed by 2 h culture in RPMI-1640 with L-glutamine (Gibco-BRL); (2)* bPEI-SPION 2 h postirradiated* antigens, which were U266 cells pretreated with 16 *μ*g/mL of bPEI-SPIONs for 1 h, followed by high-dose UVB irradiation (120 mJ/cm^2^) and 2 h culture in RPMI-1640; (3)* 16 h postirradiated* antigen composed of U266 cells, which were irradiated in the same manner as 2 h postirradiated antigen followed by overnight culture in RPMI-1640. The apoptotic cells were analyzed using the Annexin-V-FITC Apoptosis Detection Kit (BD Bioscience, San Jose, CA, USA) and pulsed with immature DCs at 2 h after maturation at a DC: apoptotic cell ratio of 2 : 1.

### 2.7. Surface Heat Shock Protein (Hsp) Expression on Tumor Antigens

FITC-Hsp70 antibody (Ab) (Cell Signaling Technology, Danvers, MA, USA) and PE-Hsp90 Ab (Abcam, Cambridge, MA, USA) were used to measure surface expression of Hsp70 and Hsp90, respectively, on tumor antigens by flow cytometry. Results represent the percentage of cells positive for expression of the total number of cells analyzed.

### 2.8. Western Blotting

Cell death mechanism of prepared antigens was evaluated by apoptotic markers such as caspase 3, cleaved caspase 3, caspase 8, cleaved caspase 8, and Bcl-XL (Cell Signaling Technology, Danvers, MA, USA) at 1 : 1000. *β*-actin (Santa Cruz Biotechnology, Dallas, Texas, USA) was used as the control marker at 1 : 500. Three types of antigen were incubated in RPMI-1640 medium with 1% P/S (10^6^ cells/10 *μ*L) for 24 h. The supernatants were collected for detection of immunogenic molecule release (Hsp70, Hsp90, and HMGB1). A total of 10 *μ*L of supernatant of each type of antigen was denatured with sodium dodecyl sulfate (SDS) (Sigma Aldrich) and loaded into one well. Primary antibodies specific to human Hsp70 (BioLegend, San Diego, USA) at 0.25 *μ*g/mL, Hsp90 (Enzo, Life Sciences, PA, USA) at 1 : 10,000, and HMGB1 (Abcam) at 1 *μ*g/mL were used.

### 2.9. Antigen Uptake Assay

To measure DC tumor antigen uptake capacity, U266 cells were stained with PKH67 (Sigma Aldrich) prior to UVB irradiation. After maturation, tumor-loaded DCs were marked with CD11c-PE (BD Bioscience). CD11c^+^DCs were analyzed using flow cytometry for expression of PKH67.

### 2.10. Phenotypic Analysis of DCs

Monoclonal antibodies against human CD80-PE, CD83-FITC, CD86-FITC, CD40-FITC, CD38-PE, CD74-FITC (BD Biosciences), and CCR7-FITC (R&D Systems, Minneapolis, MN, USA) were used to detect surface markers on DCs using flow cytometry. Cell debris was eliminated by forward and side scatter gating. The data were analyzed with WinMDI, version 2.9 (Bio-Soft Net).

### 2.11. Migration Assay

DC migration capacity was measured based on the percentage of mature DCs migrating toward CCL21 (R&D System) using a 24-well transwell plate with polycarbonate filters of 5-*μ*m pore size (Costar, Corning Incorporated, NY, USA). A total of 5 × 10^4^ DCs in 100 *μ*L of IMDM with 10% FBS and 1% P/S were added to the upper chamber, and the lower chamber contained 600 *μ*L of IMDM with 10% FBS and 1% P/S supplemented with 250 ng/mL of CCL21. The plate was incubated at 37°C for 3 h. A total of 500 *μ*L of medium in the lower chamber was collected and cells were counted for 1 minute using flow cytometry.

### 2.12. Human IL-12p70 and IL-10 Production

Mature DCs were cultured in 96-well plates at 2 × 10^4^ cells/well and stimulated with CD40L-transfected J558 cells (5 × 10^4^ cells/well), which mimics the interaction between DCs and T cells. The supernatant was collected after 24 h of coculturing. Human IL-12p70 and IL-10 cytokine levels after maturation with CD40L stimulation were determined using a BD OptEIA ELISA set (BD Biosciences).

### 2.13. Allogeneic Naïve CD4^+^ T Cell Polarization Assay

Allogeneic human T cells (2 × 10^5^ cells) isolated from PBMCs using the MACS system (Miltenyi Biotec) were cocultured with 2 h postirradiated DCs, bPEI-SPION 2 h postirradiated DCs, and 16 h postirradiated DCs (2 × 10^4^ cells) at a ratio of 5 : 1 in RPMI-1640 medium containing 10% FBS and 1% P/S. On day 5, rhIL-2 (10 U/mL, R&D Systems) was added to the T cell culture. The medium was replenished with cytokines every 2 days for 6 days. On day 11, T cells were harvested for intracellular staining. Then, 1 × 10^6^ harvested T cells were stimulated with Dynabead Human T-Activator CD3/CD28 (Gibco-BRL) for 24 h. The supernatants were collected for IFN-*γ* and IL-4 dosage by ELISA kit (BD Biosciences).

### 2.14. Intracellular Staining for Cytokine Expression

Cells were activated with 100 ng/mL of phorbol myristate acetate (PMA) and 1 *μ*g/mL of ionomycin (Sigma Aldrich) for 4 h. After 2 h of treatment with PMA and ionomycin, brefeldin A (eBioScience, San Diego, USA) was added to inhibit protein transport from the endoplasmic reticulum to the Golgi apparatus. Cells were superficially stained by CD8-APC and intracellularly marked using IFN-*γ*-FITC and IL-4-PE (BD Biosciences). Cells were analyzed by flow cytometry and data analysis was performed using Win MDI, version 2.9 (Bio-Soft Net).

### 2.15. Statistical Analysis

Results are expressed as means ± standard deviation. The difference between groups was analyzed using independent *t*-test. *P* < 0.05 was considered to indicate statistical significance.

## 3. Results

### 3.1. Optimal bPEI-SPION Concentration for Uptake by U266 Cells

**Figure 1:**
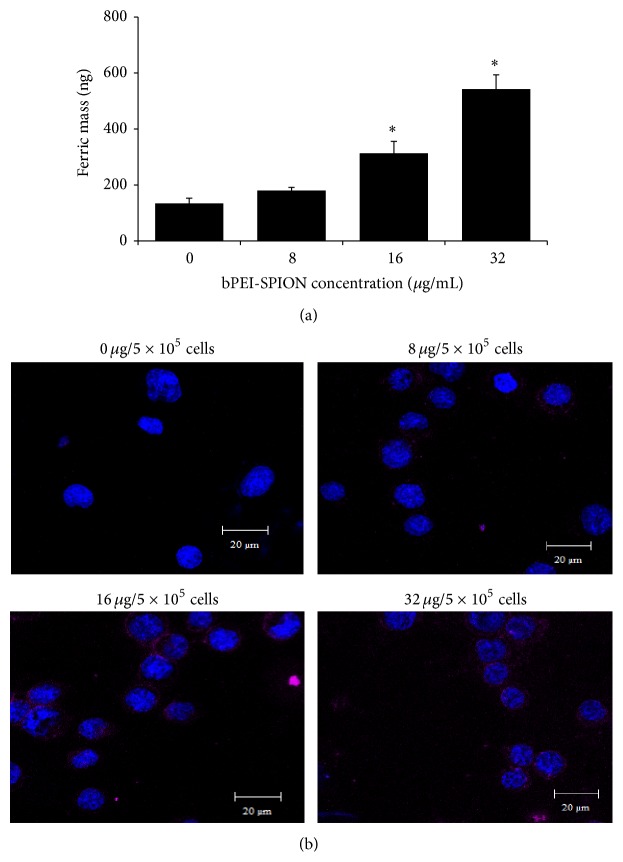
Uptake of branched polyethylenimine-superparamagnetic iron oxide nanoparticles (bPEI-SPION) by U266 cells. (a) Ferric mass contained in 5 × 10^5^ U266 cells after exposure to various amount of bPEI-SPION for 1 h. (b) The presence of nanoparticles intracellularly was confirmed by confocal microscopy in which red and blue indicate bPEI-SPION and nucleus, respectively. Data are representative of four independent experiments (^∗^ *P* < 0.001).

To optimize the concentration of bPEI-SPIONs for uptake by U266 cells, 5 × 10^5^ U266 cells were put in contact with different amount of bPEI-SPION from 0 to 32 *μ*g with shaking for 1 h at room temperature. Cells were then lysed to measure ferric mass. The ferric mass was significantly increased at bPEI-SPION amount greater than 16 *μ*g/mL (Figure 1(a)). In addition, the presence of nanoparticles inside the cells was confirmed based on confocal microscopy (Figure 1(b)). Therefore, treatment of 16 *μ*g of bPEI-SPIONs per 5 × 10^5^ cells was considered optimal for further experiments based on its cost effectiveness.

### 3.2. bPEI-SPION Pretreatment Accelerates Apoptotic Cell Death after UVB Irradiation with Induction of ROS Production

**Figure 2:**
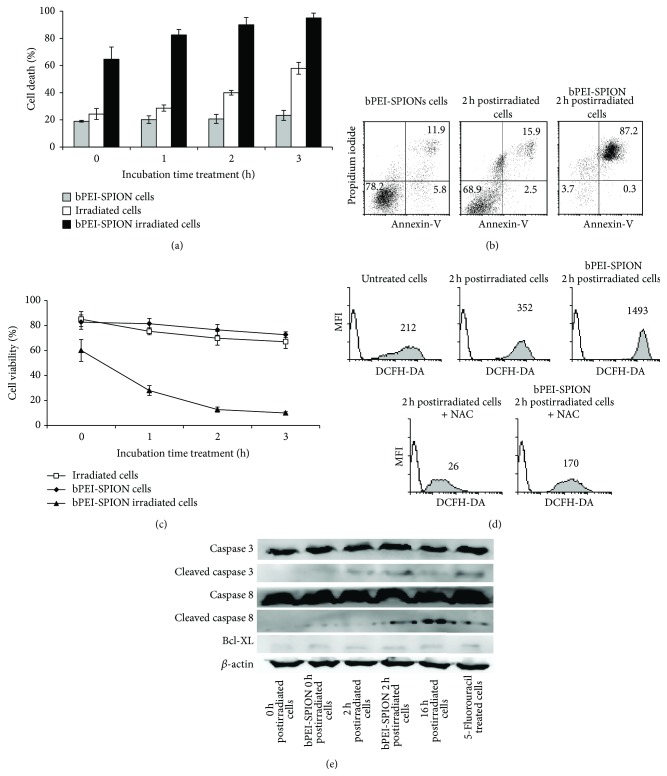
Characterization of U266 cells after UVB irradiation in the presence or absence of bPEI-SPIONs. (a) Dying U266 cells were measured using flow cytometry with propidium iodide and annexin-V. (b) bPEI-SPIONs accelerated cell death with UVB irradiation after 2 h incubation. (c) Cell viability was confirmed by Trypan blue exclusion method. (d) Intracellular reactive oxygen species (ROS) production in U266 was measured based on the fluorescence intensity of 2′,7′-dichlorofluorescein-diacetate (DCFH-DA) and confirmed using N-acetylcysteine (NAC), which blocks ROS production. bPEI-SPIONs induced high levels of ROS in U266 cells after UVB irradiation. Intracellular ROS (shaded histogram) production is indicated by the mean fluorescence intensity (MFI) compared to isotype controls (open histogram). (e) 5-Fluorouracil treated cells were used as apoptotic pathway control. bPEI-SPION 2 h postirradiated cells and 16 h postirradiated cells represented similar pattern of apoptosis markers (cleaved caspase 3, cleaved caspase 8) of control. Data are representative of three independent experiments.

After UVB irradiation, more than half of the total bPEI-SPION-pretreated cell population was in a death state, whereas cells without bPEI-SPIONs exhibited a low percentage of cell death (Figure 2(a)). In addition, 2 h after UVB irradiation, only the pretreated bPEI-SPION U266 cells showed a homogenous population in the late apoptotic/necrosis stage (Figure 2(b)). The cell viability was also confirmed with Trypan blue exclusion method (Figure 2(c)). For this reason, tumor antigen preparation was stopped 2 h after UVB irradiation. Tumor antigen ROS analysis showed that bPEI-SPIONs enhanced intracellular ROS production by up to threefold compared to nonpretreated cells, and ROS production in pretreated cells was inhibited by the antioxidant agent, NAC (Figure 2(d)). A recent report showed that SPIONs induce intracellular ROS production correlated to increase of cell death [9]. By Western blot, we showed that 2 h postirradiated cells, bPEI-SPION 2 h postirradiated cells, and 16 h postirradiated cells expressed the cleaved caspase 3 and the cleaved caspase 8 similar to cell treated with apoptosis inducer, 5-fluorouracil (Figure 2(e)). Hence, pretreated bPEI-SPION to U266 cells accelerated cell death after UVB irradiation to the late apoptotic/necrotic stage.

### 3.3. Eating-Me Signal Surface Hsp70 and Hsp90 Expression and Danger Signal Release Were Observed in 2 h Postirradiated and bPEI-SPION 2 h Postirradiated Cells

**Figure 3:**
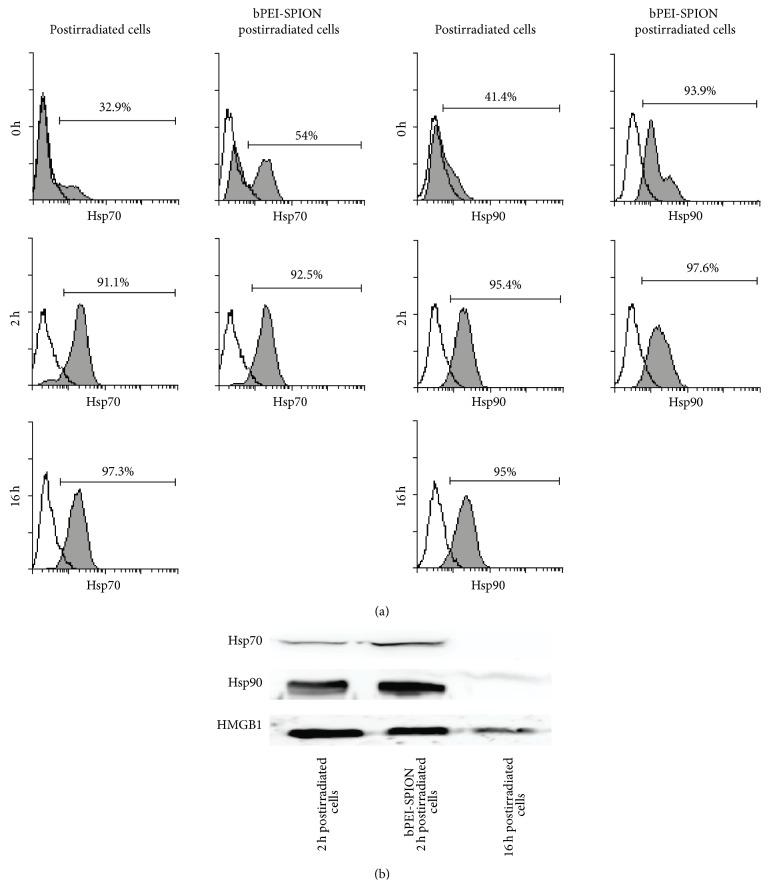
Damage-associated molecular pattern (DAMP) production by dying tumor cells induced by apoptotic pathway. (a) Expression of Hsp70 and Hsp90 on 2 h postirradiated cells, 16 h postirradiated cells, and bPEI-SPION 2 h postirradiated cells was measured by flow cytometry. Hsp70 and Hsp90 (shaded histogram) production compared with isotype controls (open histogram). 2 h postirradiated cells and bPEI-SPION 2 h postirradiated cells showed high percentage of positive cells on Hsp70 and Hsp90 expression. (b) Danger signal (Hsp70, Hsp90, and HMGB1) release from the cells was analyzed by Western blotting. bPEI-SPION 2 h postirradiated cells and 2 h postirradiated cells produced higher levels of the three danger signals compared with 16 h postirradiated cells. Data are representative of three independent experiments.

As expected, more than 90% of bPEI-SPION 2 h postirradiated cells expressed surface Hsp70 and Hsp90 (Figure 3(a)). Although 2 h postirradiated cells also showed a high percentage of Hsp70 and Hsp90 expression (Figure 3(a)), more than 60% of cells were viable (Figure 2(a)). Similarly, 16 h postirradiated cells presented high percentage of cells which were positive for these two markers. The environment in which antigens contact DCs can determine their activation status [15]. For this reason, the release of danger signals such as Hsp70, Hsp90, and HMGB1 was investigated by Western blotting. The 2 h postirradiated cells and bPEI-SPION 2 h postirradiated cells released higher levels of three danger signals compared to 16 h postirradiated cells (Figure 3(b)).

### 3.4. bPEI-SPION 2 h Postirradiated Cells Enhance Th1 Polarization without Altering DC Surface Marker Expression and DC Migration

**Figure 4:**
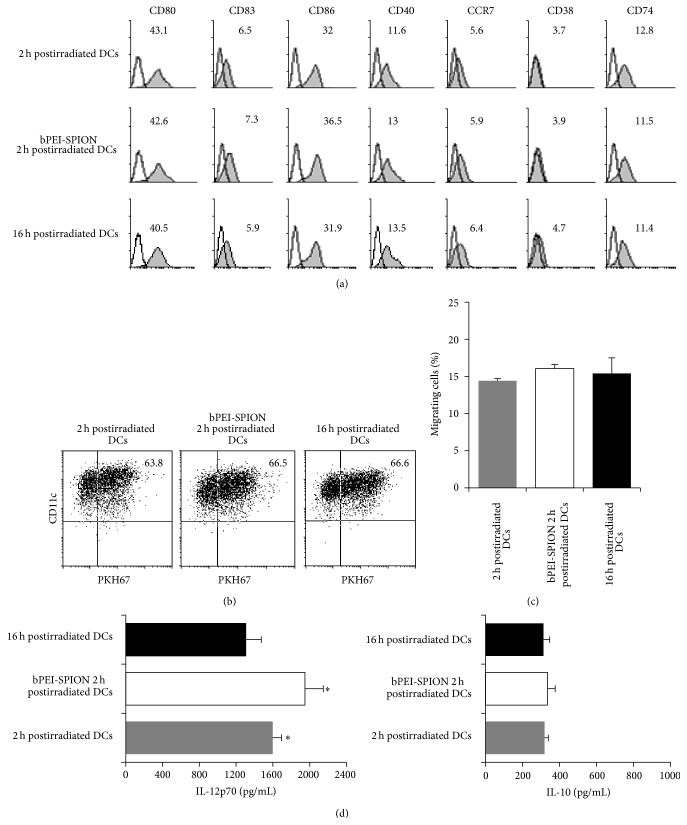
Characterization of DCs loaded with U266 cells. DCs were analyzed based on (a) flow cytometry for phenotype, (b) uptake of tumor antigens (PKH67^+^ CD11c^+^), and (c) migration capacity in response to CCL21. There were no significant differences in these parameters among the groups. (d) Cytokine production by DCs after 24-h stimulation with CD40L-transfected J558 cells was measured using ELISA. The 2 h postirradiated DCs and bPEI-SPION 2 h postirradiated DCs showed significantly higher levels of IL-12p70 secretion compared with 16 h postirradiated DCs. Data are representative of four independent experiments (^∗^ *P* < 0.05).

**Figure 5:**
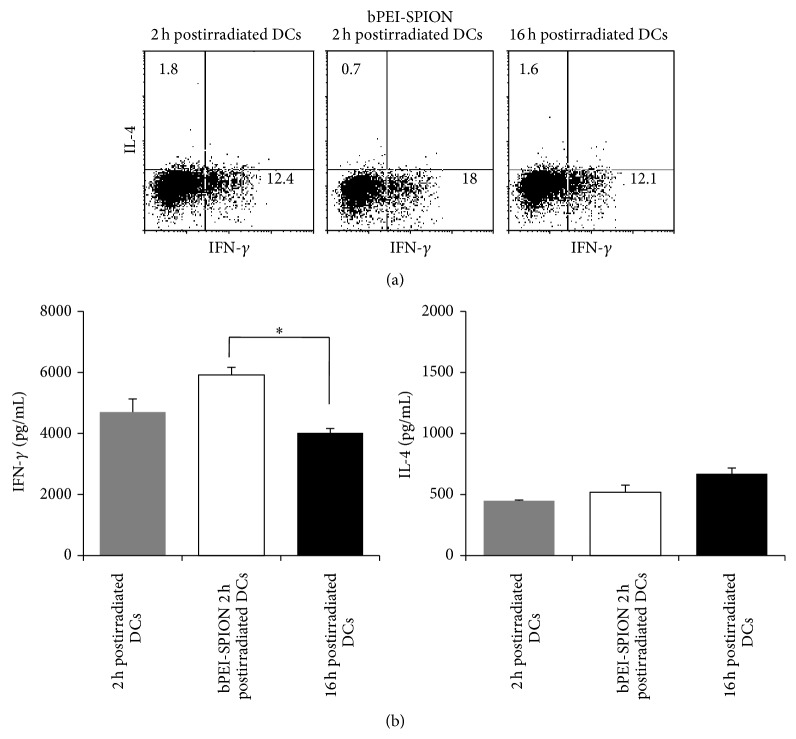
T cell polarization of DCs. Intracellular IFN-*γ* and IL-4 levels were measured using flow cytometry. bPEI-SPIONs 2 h postirradiated DCs exhibited the highest Th1 response (IL4^−^IFN*γ* ^+^) up to 18% of total T cells. (b) Amount of secreted IFN-*γ* and IL-4 was measured by ELISA. T cells polarized by bPEI-SPION 2 h postirradiated DCs presented a significant increase level of IFN-*γ*. Data are representative of three independent experiments (^∗^ *P* < 0.05).

Although danger signals were released in antigen (2 h postirradiated cells and bPEI-SPION 2 h postirradiated cells) contact milieu, surface maturation-marker expression by 2 h postirradiated DCs and bPEI-SPION 2 h postirradiated DCs was similar to that by 16 h postirradiated DCs (Figure 4(a)). In addition, there were no differences in antigen uptake and migration toward CCL21 among the groups (Figures 4(b) and 4(c)). Interestingly, 2 h postirradiated DCs and bPEI-SPION 2 h postirradiated DCs showed a significant increase in IL-12p70 production but unchanged IL-10 levels, compared with 16 h postirradiated DCs (Figure 4(d)). IL-23 production by the three types of DCs was similar (data not shown). Based on evaluation of T cell polarization by intracellular cytokine staining, bPEI-SPION 2 h postirradiated DCs showed the strongest expression of the Th1 polarizing cytokine, IFN-*γ*, reaching 18% of total analyzed T cells (Figure 5(a)). Although the IL-12p70 level produced by 2 h postirradiated DCs was higher than that by 16 h postirradiated DCs, Th1 polarization capacity was slightly increased in 2 h postirradiated DCs compared to 16 h postirradiated DCs. Besides, amount of released IFN-*γ* and IL-4 was measured by ELISA and we observed a significant increase of IFN-*γ* in T cells polarized by bPEI-SPION 2 h postirradiated DCs (Figure 5(b)).

## 4. Discussion

Recent advances have suggested that apoptosis can be immunogenic in particular death states. Upon the onset of the death stage, early apoptotic cells are characterized by an intact plasma membrane and superficial exposure of phosphatidylserine (PS) as an eating-me signal. Early apoptotic cells can progress to the late apoptotic state, also known as secondary necrotic cells, when the plasma membrane becomes permeabilized. Importantly, depending on the death state (e.g., early versus late apoptotic), recognition and internalization by phagocytes can result in an anti- or proinflammatory response [5, 16]. We showed here that bPEI-SPIONs accelerated cell death through apoptotic pathway after UVB irradiation with increasing intracellular ROS production and induced extracellular release of Hsp70, Hsp90, and HMGB1. Increases in intracellular ROS give rise to high level expression of several Hsps to protect cells from damage [17]. In this study, considerable quantities of Hsp70, Hsp90, and HMGB1 were released from 2 h postirradiated cells and bPEI-SPION 2 h postirradiated cells. We hypothesized that UVB irradiation causes only mild damage to cells; hence, part of the cell population entered the death stage, resulting in gradual damage signal accumulation. On the other hand, excessive cell damage occurred in the bPEI-SPION-pretreated cell population, which caused simultaneous death; hence the damage signals were released immediately. Therefore, bPEI-SPION 2 h postirradiated cells were the most immunogenic tumor antigens [6]. In literature, danger signals have been reported to DC maturation stimuli [18]. Our results indicated release of at least three danger signals—Hsp70, Hsp90, and HMGB1—from tumor antigens. Even so, these stimuli were not sufficient to change DC surface marker expression. However, Hsp and HMBG1 effectively promoted T cell polarization to the Th1 subset. This result was in agreement with previous findings regarding the effect of Hsp and HMGB1 on Th1 polarization [19, 20].

In conclusion, bPEI-SPIONs, which are nontoxic elements, accelerate tumor cell death to the immunogenic late apoptotic/necrosis stage after a short incubation after UVB irradiation and can serve as an antigen for loading onto DCs. DCs loaded with the antigen exhibited basic properties of DCs loaded with 16 h postirradiated cells with high Th1 polarization characteristics. These results suggest that bPEI-SPION 2 h postirradiated cells are useful and promising tumor antigens in DCs for induction of antigen-specific T cell immune responses against MM.
